# Editorial: Addictive disorders and digital medicine: technology-based solutions for addictive disorders

**DOI:** 10.3389/fpsyt.2025.1674826

**Published:** 2025-09-03

**Authors:** Sang-Kyu Lee, Chul-Hyun Cho, Daniel L. King

**Affiliations:** ^1^ Department of Psychiatry, College of Medicine, Hallym University Medical Center, Chuncheon, Republic of Korea; ^2^ Department of Psychiatry and Biomedical Informatics, Korea University College of Medicine, Seoul, Republic of Korea; ^3^ College of Education, Psychology, & Social Work, Flinders University, Adelaide, SA, Australia

**Keywords:** addictive disorder, digital therapeutics, technology-based intervention, artificial intelligence, mental health innovation

Addictive disorders pose a major global health burden, yet access to effective treatment remains limited (1). This Research Topic brings together twelve original contributions that showcase how digital innovations—ranging from mobile applications and blended interventions to AI-driven tools—are reshaping addiction care (2). The contributions span four main domains: (1) mobile and app-based interventions, (2) blended or hybrid digital therapies, (3) artificial intelligence and immersive technologies, and (4) psychosocial and neurocognitive correlates of digital addiction.

Mobile and app-based solutions highlight the scalability and personalization potential of digital CBT (3). Redeł et al. developed Nałogometr 2.0, a mobile app incorporating CBT and mindfulness elements with real-time ecological assessments. Jeong et al. evaluated a digital self-care device that uses behavioral metrics to assess alcohol-related risk. Lim et al. demonstrated the superiority of digital CBT over face-to-face CBT in improving abstinence rates and engagement.

Several studies explored hybrid models that blend digital and traditional therapies. Tarp et al. examined therapist experiences with Blend-A, showing successful clinical integration despite initial resistance. Schettini et al. analyzed gender differences in iCBT outcomes for alcohol use disorder, suggesting broad applicability. Labrenz et al. targeted adolescents with digital media use disorder through a blended mobile and group therapy model, while Meads et al. assessed a wearable neuromodulation device for opioid recovery, showing feasibility and improved sleep.

AI and virtual platforms are emerging as transformative tools. Joseph et al. discussed the promise of machine learning for real-time, adaptive interventions in underserved populations. Lee et al. provided a systematic review of chatbot-based interventions, with strong results for smoking cessation. Matthews et al. proposed virtual reality psychedelic simulations (VRP) for alcohol use disorder, positioning VRP as a complementary or standalone therapeutic tool.

Two studies expanded the conversation to include emotional and neurocognitive vulnerabilities underlying digital addiction. Ursoniu et al. linked social media addiction to alexithymia and empathy deficits in medical students. Liu et al. used network analysis to show that autistic traits, communication difficulties, and cyberspace-oriented relationships are key nodes connecting digital addiction and depression among college students. These findings call for more nuanced, trans-diagnostic intervention strategies.

## Conclusion and future directions

This Research Topic illustrates the multifaceted potential of digital solutions in addiction care from enhancing access and engagement to tailoring interventions through machine learning. Just-in-time adaptive interventions (JITAI), wearable biosensors for craving detection, and chatter-bot based therapy represent a new frontier. Future research should prioritize real-world implementation, cross-platform integration, and user-centered design, especially for marginalized and high-risk populations. We hope this Research Topic encourages interdisciplinary collaboration and accelerates the integration of digital medicine into mainstream addiction treatment.

## References

[1] WHO. Digital health and addiction services: Global strategy brief. Geneva: World Health Organization (2023).

[2] Linardon J Shatte A. eMental Health interventions for comorbid depression and substance use. J Affect Disord. (2017) 221:393–404. doi: 10.1016/j.jad.2017.06.003, PMID: 28628763

[3] Wang Y Smith C Zhao L KIm J Patel R Nguyen H. AI-based craving detection using wearable biosensors. Addict Biol. (2022) 27:e13124. doi: 10.1111/adb.13124, PMID: 34894025

